# Mechanisms Underlying Phase Transition and Regulation of Tantalum Powder Properties During Magnesium Thermal Reduction of Ta_2_O_5_ in a Molten Salt Medium

**DOI:** 10.3390/ma18051115

**Published:** 2025-03-01

**Authors:** Yi Chen, Zhenghao Han, Tianchen Li, Ruifang Wang, Chao Zhang, Yusi Che, Jilin He

**Affiliations:** 1 School of Material Science and Engineering, Zhengzhou University, Zhengzhou 450001, China; cyclxy@gs.zzu.edu.cn (Y.C.); hzh20000826@zzu.edu.cn (Z.H.); s1401487138@gs.zzu.edu.cn (T.L.); wangruifang@zzu.edu.cn (R.W.); hejilin@zzu.edu.cn (J.H.); 2 Zhongyuan Critical Metals Laboratory, Zhengzhou University, Zhengzhou 450001, China; 3 School of Thermal Engineering, Shandong Jianzhu University, Jinan 250101, China; zhangchao19@sdjzu.edu.cn

**Keywords:** magnesium reduction, tantalum powder, tantalum pentoxide, reaction pathway, phase transformation mechanisms

## Abstract

Magnesium reduction of Ta_2_O_5_ (tantalum pentoxide) is a metallurgical process widely used to extract metallic tantalum powder from its oxide form using magnesium as a reducing agent in a molten salt medium. This study explores the mechanisms and patterns of phase transformation during the magnesium reduction of Ta_2_O_5_ in a molten salt medium, focusing on the influence of temperature and time on the physical and chemical properties of the resulting tantalum powder. The results reveal that under various reaction conditions in a molten salt medium, the magnesium reduction of Ta_2_O_5_ follows four distinct pathways: Ta_2_O_5_ → Ta, Ta_2_O_5_ → MgTa_2_O_6_ → Ta, Ta_2_O_5_ → MgTa_2_O_6_ → Mg_4_Ta_2_O_9_ → Ta, and Ta_2_O_5_ → Mg_4_Ta_2_O_9_ → Ta. Each pathway significantly affects the physical and chemical properties of the resulting tantalum powder. Using a uniform mixing method, the reaction proceeds directly from Ta_2_O_5_ to Ta powder in a single step. As the reaction temperature increases from 600 °C to 900 °C, the average particle size of the tantalum powder enlarges from 30 nm to 150 nm, with the product phase transitioning from a mixture of Ta and Ta_2_O to a single Ta phase. Additionally, prolonged holding time improves the uniformity of the tantalum powder’s particle distribution. This study accomplishes directional control over the phase transformation and the properties of tantalum powder during the reduction process, thus offering valuable guidance for the preparation of high-performance tantalum powder.

## 1. Introduction

Tantalum, a strategic critical metal, is known for its high density, high melting point, and high thermal conductivity [1]. As a valve metal, tantalum forms a stable oxide film on its surface, providing excellent corrosion resistance and a high dielectric constant. Owing to these unique properties, it is widely used in industries such as chemical engineering, electronics, steel smelting, aerospace, and biomedical applications [2,3,4]. The primary use of tantalum is in the production of tantalum capacitors, which account for approximately 50–60% of its consumption [5,6,7]. Compared to other types, tantalum capacitors offer advantages such as large capacity, compact size, wide operating temperature range, and high reliability, making them indispensable in electronic devices, communication equipment, military applications, and aerospace industries [8,9,10,11].

The performance of tantalum capacitors is directly influenced by the physical and chemical properties of tantalum powder, which are closely related to its preparation process. The tantalum powder can be synthesized through various methods, including sodium reduction of potassium fluorotantalate [12,13,14], magnesium reduction of tantalum oxide [15,16,17], carbothermal reduction [18,19,20], FFC electrolysis [21,22], chloride reduction [23,24], and homogeneous reduction [25]. Of these methods, only the sodium reduction of potassium fluorotantalate and the magnesium reduction of tantalum oxide have been industrialized. Currently, the sodium reduction method is widely used, where potassium fluorotantalate is reduced using liquid sodium as the reducing agent in the presence of a halide salt diluent, producing tantalum powder with a relatively high specific surface area. However, this method has limitations of a lengthy process, difficulty in controlling the reaction, uneven particle size of tantalum powder, high impurity content, and environmental concerns due to the toxic and harmful fluorides produced, making it unsuitable for meeting the high-pressure and high-reliability requirements of tantalum capacitors [26,27,28].

For the synthesis of metallic tantalum powder, researchers have mainly used gaseous magnesium reduction of tantalum oxide and the magnesiothermic self-propagating method. Shekhter et al. [29,30]. conducted extensive research on the preparation of tantalum powder via gaseous magnesium reduction of tantalum oxide and found challenges of controlling magnesium vapors during the reaction process and imposing stringent equipment requirements, which make it difficult to ensure a homogeneous reaction environment, thereby limiting its widespread application. Hwang et al. discovered that in the gaseous magnesium reduction process, tantalum oxide undergoes a transition from Ta_2_O_5_ to Ta_2_O to Ta, where single oxide particles are gradually reduced from the outside in [15]. In 1998, H.C. Starck, a German company, began researching gaseous magnesium reduction tantalum powder preparation technology and realized mass production in 2006, monopolizing the global high-pressure, high-capacitance tantalum powder market. The developed magnesium-reduced tantalum powder particles are uniform and the formation voltage can be up to 400 V. In the same excitation voltage, the magnesium-reduced tantalum powder has a higher specific capacitance than the sodium-reduced tantalum powder by roughly 20–30%, which is of significant superiority [31,32], while the domestic has not yet overcome the technology [33]. Professor Zhang’s team at Northeastern University found that the magnesiothermic self-propagating method is a highly exothermic reaction, leading to rapid and incomplete reduction reactions, resulting in low-valent oxides and composite oxides in the product that cannot be easily removed by wet processing [34]. Orlov et al. reported that magnesium tantalate often forms during the magnesiothermic self-propagating process and tantalum powder derived from magnesium tantalate reduction has a larger specific surface area than that obtained from direct tantalum oxide reduction [35].

In order to produce high-performance tantalum powder with controllable particle size and uniform particles, based on these methods, for the difficult problem of difficult to regulate the magnesium thermal reaction process, our team, in collaboration with Ningxia Orient Tantalum Industry Co., Ltd. (Shizuishan, China), proposed and developed a method of magnesium reduction of tantalum oxide in a molten salt medium. This method effectively absorbs the exothermic reactions’ heat, offering advantages in reaction control and powder properties regulation.

At present, due to the lack of relevant basic research, the evolution of the physical phase in the process of magnesium reduction of tantalum oxide is not clear, which causes great difficulties in the removal of oxides in tantalum powder and the regulation of uniformity [34,35]. In this paper, the production of tantalum powder by reduction of tantalum oxide using magnesium as a reducing agent in a molten salt medium is studied, and the reduction rate is controlled by varying material distribution methods. This research systematically explores phase evolution during the magnesium reduction of tantalum oxide, playing a crucial role in understanding the magnesium reduction mechanism and controlling the reduction process and the final properties of the resulting tantalum powder.

## 2. Experimental Section

### 2.1. Materials

**Figure 1:**
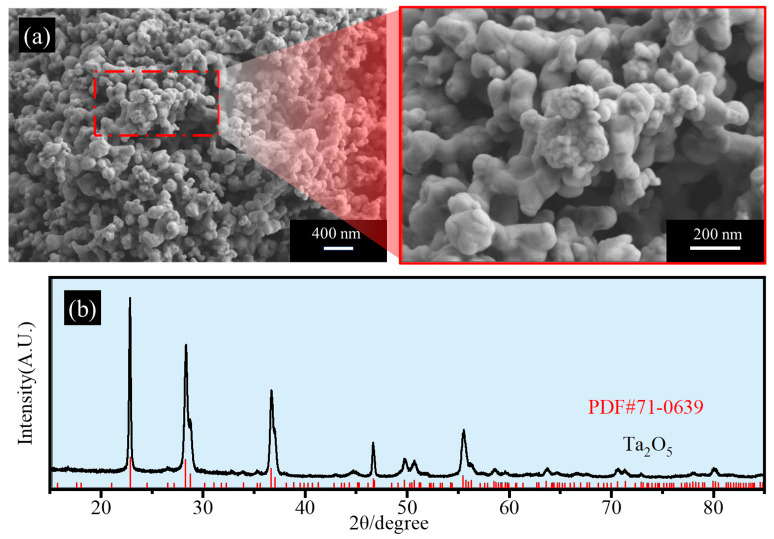
SEM-EDS results (**a**) and XRD patterns (**b**) of experimental tantalum pentoxide.

In this study, tantalum pentoxide (≥99.5%; Jiujiang Nonferrous Metals Smelting Co., Ltd., Jiujiang, China) was used as the raw material, magnesium metal (99.8%; Tangshan Weihe Magnesium Powder Co., Ltd., Tangshan, China) as the reducing agent, and potassium chloride (99.8%; Shanghai Macklin Biochemical Technology Co., Ltd., Shanghai, China) as the diluent. The microstructure and composition analysis of tantalum oxide, as depicted in Figure 1, reveal that the primary phase of the raw material is Ta_2_O_5_, with particle diameters ranging from 150 to 300 nm. These particles are bonded together, forming a honeycomb-like agglomerated structure.

The phase composition of the raw materials and reaction products was analyzed using an X-ray diffractometer (XRD) (PANalytical Empyrean, Almelo, The Netherlands) with a step size of 0.02° over the 2θ range of 10–90°. The oxygen content in the products was determined by an oxygen/nitrogen/hydrogen analyzer (NCS, Beijing, China), employing a pulse heating-infrared absorption method with ppm-level accuracy. The microstructure and elemental composition of the raw materials and synthesized products were analyzed using scanning electron microscopy (SEM) (ZEISS, Oberkochen, Germany) equipped with an energy-dispersive spectroscopy (EDS) system (resolution: 0.8 nm). Particle size distribution of tantalum powder was analyzed after 10 min of ultrasonic dispersion using a laser particle size analyzer (Malvern, Malvern, UK) with a measuring range of 0.02–2000 μm.

### 2.2. Methods

**Figure 2:**
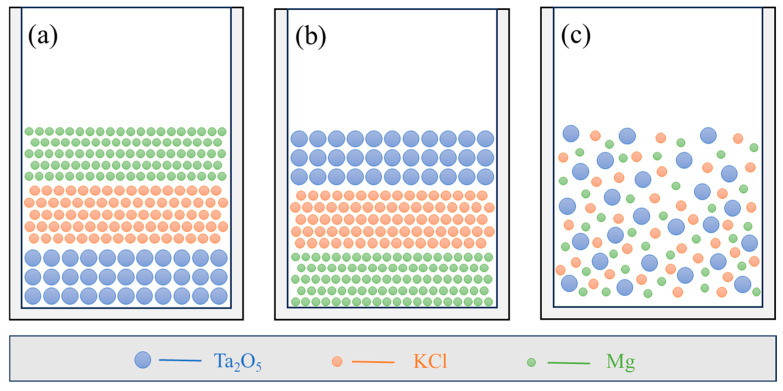
Spreading of raw materials in the crucible; (**a**) Tantalum pentoxide on the bottom; (**b**) Tantalum pentoxide on the top; (**c**) homogeneous mixture.

To control the reaction rate and analyze intermediate phases, three distinct material placement methods were designed, as illustrated in Figure 2. In placement method A, the crucible is sequentially layered from top to bottom with magnesium powder, potassium chloride, and tantalum pentoxide (Figure 2a). In placement method B, the lower section of the crucible contains magnesium powder, the upper layer contains tantalum pentoxide, and potassium chloride is placed between them (Figure 2b). In placement method C, all three raw materials are uniformly mixed together (as shown in Figure 2c).

Using three distinct material placement methods, specific amounts of magnesium, potassium chloride, and tantalum pentoxide were weighed and loaded into an alumina crucible (30 mm in diameter and 60 mm in height). The alumina crucible containing the materials was then placed into a tube furnace. The tube furnace was evacuated multiple times and subsequently heated in an argon atmosphere at a rate of 10 °C/min to a predetermined temperature, where it was held from 0 to 5 h. After the holding time, the furnace was cooled to room temperature.

The product was thoroughly washed with distilled water (multiple times) to remove potassium chloride from the reduction products. The washed solid product was then filtered and separated, followed by stirring and soaking in a 10% nitric acid solution for 1 h to eliminate magnesium oxide impurities. Finally, the acid-washed solid product was rinsed with distilled water until the rinse water was neutral and then dried at 60 °C for 12 h.

## 3. Results and Discussion

### 3.1. Thermomechanical Analysis

**Figure 3:**
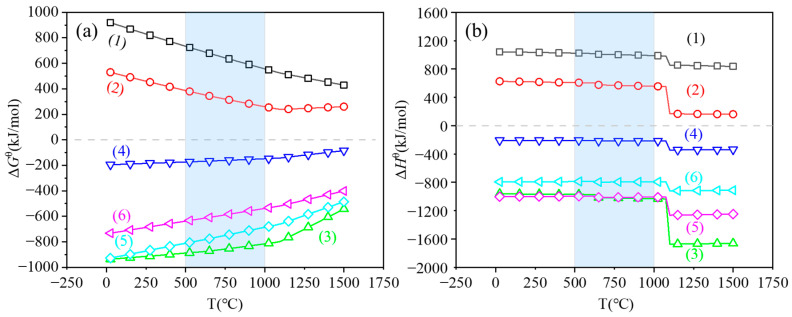
Standard Gibbs free energy change (**a**) and standard molar enthalpy change (**b**) for the magnesium reduction of tantalum oxide as a function of temperature.

Due to the multiple valence states of tantalum, various chemical reactions can occur during the magnesium reduction of tantalum pentoxide, as expressed in Equation (1) through (6). The standard Gibbs free energy change and standard enthalpy change for these reactions were calculated at different temperatures using the reaction module of the thermodynamic calculation software Factsage 8.3, as summarized in Figure 3. Figure 3a shows that in a temperature range of 25 to 1500 °C, both tantalum pentoxide and the lower valence tantalum oxides (TaO_2_ and TaO) can be reduced to tantalum, whereas the reduction of Ta_2_O_5_ to TaO_2_ and TaO does not occur spontaneously. As the temperature increases, the standard Gibbs free energies of magnesium reduction of tantalum oxides of different valence states are closer to the 0 scale; the lower reaction temperatures are favorable for the reduction reaction.

(1) $$Ta_{2}O_{5}+\mathrm{Mg}=2\mathrm{Ta}O_{2}+\mathrm{MgO}$$

(2) $$Ta_{2}O_{5}+3\mathrm{Mg}=2\mathrm{TaO}+3\mathrm{MgO}$$

(3) $$Ta_{2}O_{5}+5\mathrm{Mg}=2\mathrm{Ta}+5\mathrm{MgO}$$

(4) $$\mathrm{Ta}O_{2}+\mathrm{Mg}=\mathrm{TaO}+\mathrm{MgO}$$

(5) $$\mathrm{Ta}O_{2}+2\mathrm{Mg}=\mathrm{Ta}+2\mathrm{MgO}$$

(6) TaO+Mg=Ta+MgO

Figure 3b shows that the standard enthalpy values for reactions (3) through (6) are negative, indicating that these reactions are exothermic in nature. As the temperature rises, the energy states of the reactants and products change, leading to increased exothermic heat. To improve reaction efficiency and mitigate the negative effects of uneven tantalum powder growth caused by the exothermic reaction, this study comprehensively investigates the magnesium reduction of tantalum pentoxide at temperatures ranging from 500 to 1000 °C.

**Figure 4:**
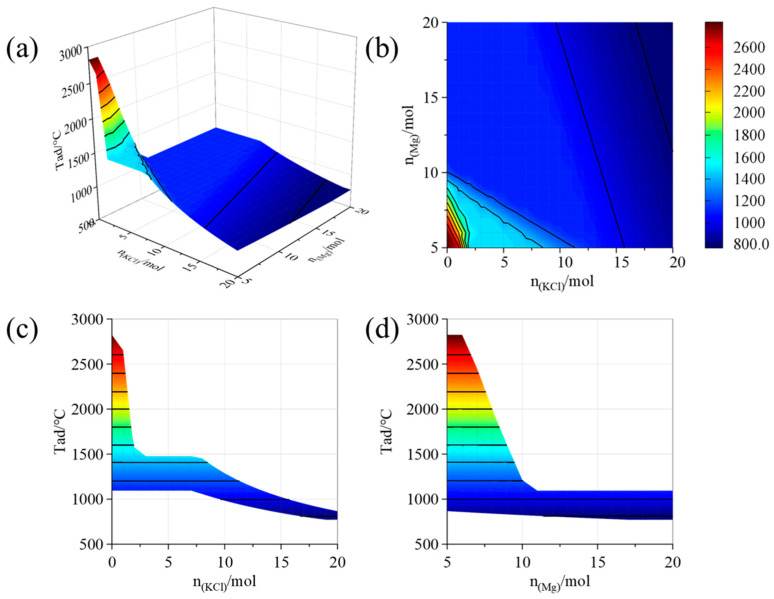
Effect of content of magnesium and potassium chloride on the adiabatic temperature of the system: (**a**) 3D surface view; (**b**) top view; (**c**) main view; and (**d**) side view.

Additionally, the effect of potassium chloride and magnesium content on the adiabatic temperature of the Ta_2_O_5_-Mg-KCl system was analyzed using Factsage, as illustrated in Figure 4.

When the potassium chloride content is zero and the magnesium content is present at the theoretical amount, the system’s adiabatic temperature reaches its highest value of 2825 °C, which is the melting point of magnesium oxide. As the potassium chloride content increases with the magnesium, the system’s maximum adiabatic temperature gradually decreases, due to the addition of a lower melting point constituent. Due to the latent heat of phase transitions, two significant temperature plateaus occur at the boiling points of potassium chloride (1475 °C) and magnesium (1095 °C). Notably, at the boiling point of magnesium, changes in the adiabatic temperature of the reaction system occur only with substantial variations in the amounts of magnesium and potassium chloride. Considering the adiabatic temperature and the amounts of magnesium and potassium chloride, a system with an adiabatic temperature of 1095 °C was selected for the study of magnesium reduction process in detail.

### 3.2. Evolution of the Physical Phase During Magnesium Reduction

**Figure 5:**
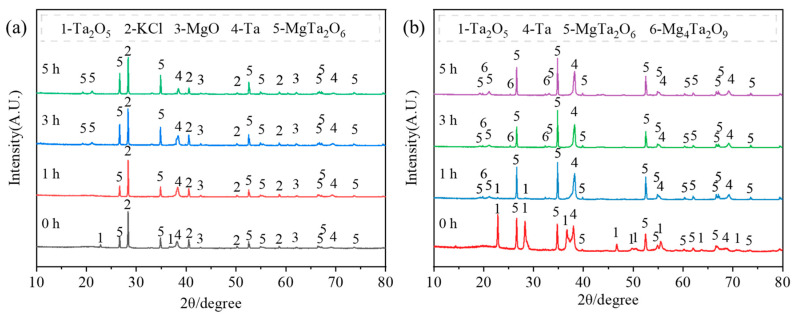
XRD patterns of the products before and after acid washing (placement method A). (**a**) after reduction; (**b**) after acid washing.

The phase evolution at 900 °C was investigated under different material placement methods and holding times. Figure 5 presents the phase composition of the primary reduction products and the acid-washed products under placement method A.

As shown in the XRD patterns in Figure 5a, extending the holding time gradually eliminates the Ta_2_O_5_ phase in the product, and after 5 h, the developed phases include Ta, MgTa_2_O_6_, KCl, and MgO. A comparison of the products before and after acid washing under the same conditions reveals that KCl and MgO are washed out in acid, while Mg_4_Ta_2_O_9_ emerges along with existing MgTa_2_O_6_. This indicates that after KCl dissolves in water and MgO dissolves in acid, the relative content of Mg_4_Ta_2_O_9_ increases, leading to its stronger diffraction peaks. It also suggests that tantalum oxides such as Mg_4_Ta_2_O_9_, MgTa_2_O_6_, and Ta_2_O_5_ are insoluble in dilute nitric acid. In Figure 5b, it is evident that without holding at 900 °C, the reduction products consist of Ta, MgTa_2_O_6_, and Ta_2_O_5_, where a part of the tantalum oxide is reduced to tantalum, and magnesium oxide combines with unreacted Ta_2_O_5_ to form MgTa_2_O_6_. In placement method A, the limited contact between magnesium and tantalum oxide slows the reaction rate, leaving a significant amount of tantalum oxide unreduced even after 5 h. The formation of magnesium tantalate can be explained by the possible reactions expressed in Equations (7)–(9) [36,37,38]. Due to the lack of thermodynamic data for magnesium tantalate, the feasibility of these reactions cannot be verified through thermodynamic calculations. However, it can be shown by Baskin et al. that the Mg_4_Ta_2_O_9_ and MgTa_2_O_6_ phases can exist stably, and the two intermediate phases of magnesium tantalate produced during the reduction process in this paper are stable phases [39].

(7) $$Ta_{2}O_{5}+\mathrm{MgO}=\mathrm{MgT}a_{2}O_{6}$$

(8) $$Ta_{2}O_{5}+4\mathrm{MgO}=Mg_{4}Ta_{2}O_{9}$$

(9) $$\mathrm{MgT}a_{2}O_{6}+3\mathrm{MgO}=Mg_{4}Ta_{2}O_{9}$$

**Figure 6:**
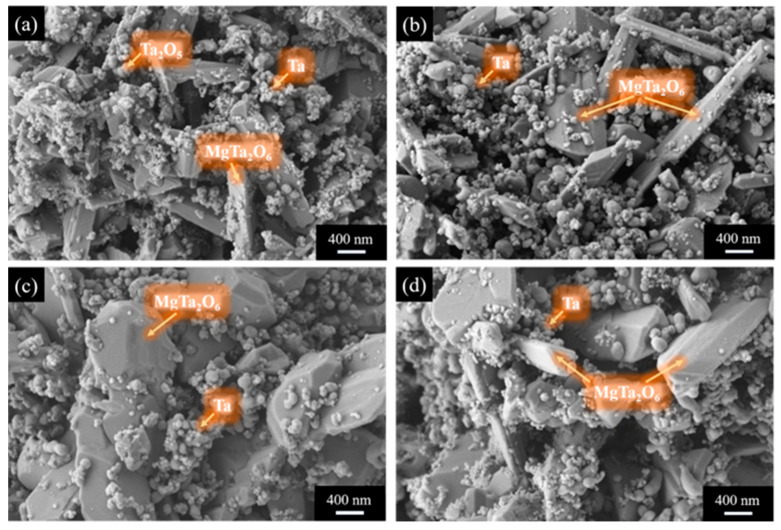
SEM images showing morphology of pickling products (of placement method A) at different holding time conditions: (**a**) 0 h; (**b**) 1 h; (**c**) 3 h; and (**d**) 5 h.

Figure 6 shows the SEM microstructure of the acid-washed products obtained at different holding times for placement method A. The EDS analysis of the products under different conditions reveals that at 900 °C without any holding time, the acid-washed products consist of large plate-like MgTa_2_O_6_, smaller particles of tantalum, and tantalum oxide, with the tantalum obtained after reduction largely retaining the original morphology of the Ta_2_O_5_. As the holding time increases, the magnesium tantalate grows to some extent, while the tantalum powder particles show minor changes.

**Figure 7:**
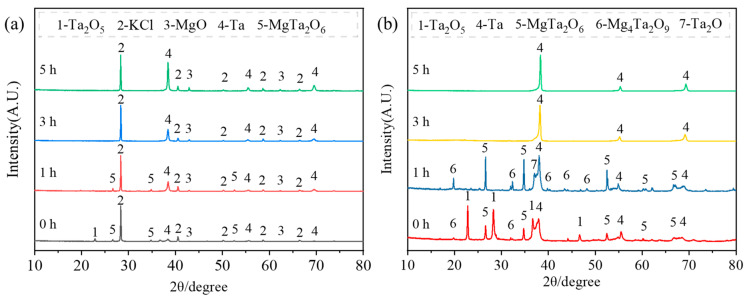
XRD patterns of the products before and after acid washing (placement method B). (**a**) after reduction; (**b**) after acid washing.

Figure 7 presents the crystallinity and phase composition of the products obtained under different holding times for placement method B. Similar to placement method A, the water and acid washing effectively remove magnesium oxide and potassium chloride. Without holding time, the reduction products consist of unreacted Ta_2_O_5_, Ta, MgTa_2_O_6_, and Mg_4_Ta_2_O_9_. When the holding time is increased to 1 h, all Ta_2_O_5_ converts to Ta, Mg_4_Ta_2_O_9_, and MgTa_2_O_6_, with Mg_4_Ta_2_O_9_ being the product of a further combination of tantalum oxide and magnesium oxide. The presence of Ta_2_O after washing suggests slight surface oxidation of Ta during the acid-washing process. Extending the holding time beyond 3 h results in only the tantalum phase remaining, indicating that magnesium tantalates (Mg_4_Ta_2_O_9_ and MgTa_2_O_6_) can also be reduced to tantalum, as indicated by the possible reactions in Equations (10) and (11) [35,40].

(10) $$\mathrm{Mg}{\mathrm{Ta}}_{2}O_{6}+5\mathrm{Mg}=2\mathrm{Ta}+6\mathrm{MgO}$$

(11) $$Mg_{4}Ta_{2}O_{9}+5\mathrm{Mg}=2\mathrm{Ta}+9\mathrm{MgO}$$

Compared to placement method A, method B reveals higher reduction efficiency. It is conceived that in method B, tantalum pentoxide particles easily move downward to contact magnesium after passing through the molten salt layer, whereas in method A, liquid magnesium, hindered by surface tension, struggles to penetrate the molten salt layer to react with tantalum pentoxide, resulting in losses due to vaporization.

**Figure 8:**
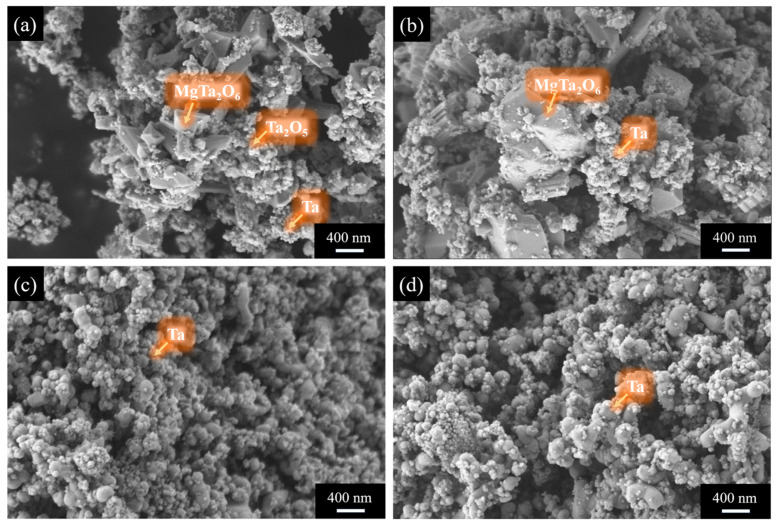
SEM images showing morphology of pickling products (of placement method B) at different holding time conditions: (**a**) 0 h; (**b**) 1 h; (**c**) 3 h; and (**d**) 5 h.

Figure 8 presents the microstructure of the products obtained under different holding times for placement method B. Unlike method A, where MgTa_2_O_6_ appears in large, plate-like structures, MgTa_2_O_6_ in method B is blocky and smaller in size. Additionally, in method B, Ta_2_O_5_ and Ta particles have similar sizes. When the holding time exceeds 3 h, both magnesium tantalate phases are completely reduced to tantalum, and the size of the reduced tantalum powder particles remains largely unaffected by a further increase in holding time.

**Figure 9:**
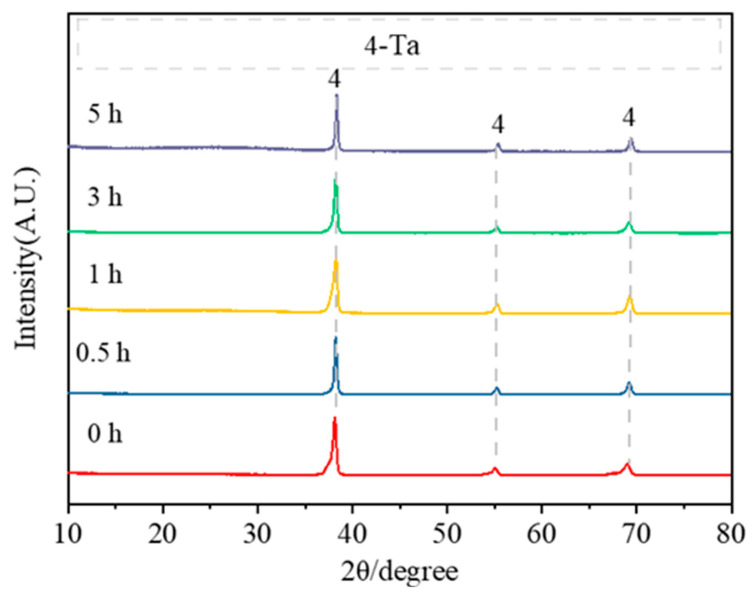
XRD patterns of the products obtained after acid washing (in placement method C).

Figure 9 illustrates the phase transition at 900 °C with different holding times under placement method C. At this temperature, the products consist solely of the pure metallic tantalum phase, indicating that the tantalum pentoxide has been fully reduced with no intermediate phases observed, suggesting that the intermediate phase is unstable or does not have the conditions for formation in this reduction method. Investigation into these possible mechanisms is ongoing.

**Figure 10:**
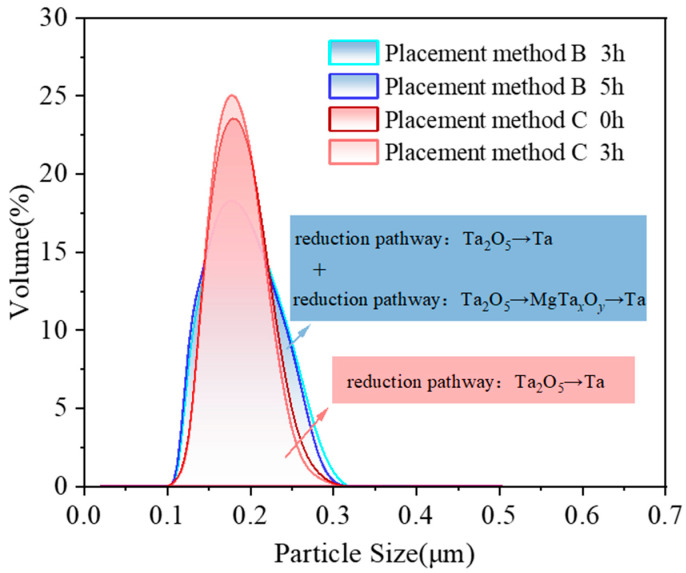
Comparison of particle size distribution (measured using Laser particle size analyzer) of pure metallic tantalum powder synthesized at 900 °C under different holding times, in placement methods B and C.

Figure 10 and Table 1 summarize a comparison of the particle size distribution characteristics of tantalum powder obtained at 900 °C under different holding times for placement methods B and C. As in method B, a minimum of 3 h is required for a stable Ta phase formation (however, a minimum of 0 h is sufficient in case of uniform mixing (method C)); therefore, it is compared the particle size distribution of Ta powder successfully synthesized in methods B and C at different holding times.

The *SPAN* value calculated using Equation (12) represents the uniformity of the tantalum powder particle size, and the smaller *SPAN* value represents the more uniform powder size.

(12) $$\mathrm{SPAN}=\frac{D_{90}-D_{10}}{D_{50}}$$

In the equation, *D*_10_, *D*_50_, and *D*_90_ represent the particle sizes at which 10%, 50%, and 90% of the total powder volume, respectively, have a particle size smaller than these values, with the units being micrometers (μm).

As is evident from the figures and tables, under the same holding time, the *SPAN* value of tantalum powder obtained under uniform mixing conditions is 0.46, which is significantly smaller than 0.60 when magnesium is placed in the lower layer, indicating a more concentrated particle size distribution. When magnesium is in the lower layer, multiple phase evolution pathways exist (Ta_2_O_5_ → Ta and Ta_2_O_5_ → MgTa*_x_*O*_y_* → Ta), resulting in more small and large particles, which leads to an uneven particle size distribution. At the same temperature, by extending the holding time, the *SPAN* value of tantalum powder under uniform mixing conditions decreases from 0.46 to 0.43, and the *SPAN* value of tantalum powder obtained by reduction with magnesium in the lower layer decreases from 0.60 to 0.57. This results in a more concentrated particle size distribution and improved uniformity of the tantalum powder, although the change in particle size is relatively small. The increase in tantalum powder size mainly depends on the nucleation and growth mechanisms during the reduction process.

**Figure 11:**
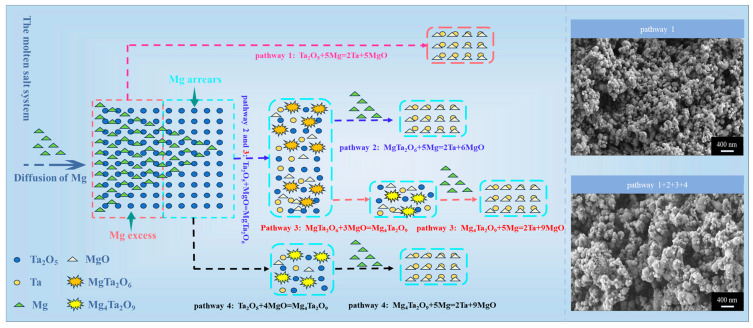
Principle of phase transition during magnesium reduction of tantalum pentoxide. Different routes highlight the diffusion of magnesium for complete reduction of Ta_2_O_5_ to Ta.

Based on the experimental results, the phase transformation patterns during the magnesium reduction of tantalum pentoxide are summarized as illustrated in Figure 11. As magnesium diffuses to contact Ta_2_O_5_, uneven diffusion creates regions with varying concentrations, some with an excess and others with a deficiency. In regions with excess magnesium, Ta_2_O_5_ is completely reduced to Ta and MgO, whereas in magnesium-deficient regions, only part of Ta_2_O_5_ is reduced to tantalum, and unreacted Ta_2_O_5_ combines with MgO to form magnesium tantalate (MgTa_2_O_6_ and Mg_4_Ta_2_O_9_). As magnesium continues to diffuse, sufficient magnesium in these regions allows the further reduction of magnesium tantalate and any remaining Ta_2_O_5_ to tantalum. Considering the formation of magnesium tantalate, four possible reaction pathways during the magnesium reduction of tantalum oxide are shown in Figure 11: Ta_2_O_5_ → Ta; Ta_2_O_5_ → MgTa_2_O_6_ →Ta; Ta_2_O_5_ → MgTa_2_O_6_ → Mg_4_Ta_2_O_9_ → Ta; and Ta_2_O_5_ → Mg_4_Ta_2_O_9_ → Ta.

Notably, all four reaction pathways are observed in placement methods A and B, whereas placement method C is dominated exclusively by pathway 1 (Ta_2_O_5_ → Ta). According to the findings of Orlov et al., ensuring the consistency of the reaction pathway in the magnesium reduction process is crucial for achieving a uniform particle size distribution of tantalum powder [35,41]. In placement methods A and B, both tantalum powder obtained from the direct reduction of tantalum oxide and the reduction of magnesium tantalate contribute to the non-uniformity of particle size. In contrast, placement method C, which is free from these additional influences, is more effective in ensuring the uniformity of the resulting tantalum powder.

### 3.3. Effect of Reduction Temperature and Time on the Physicochemical Properties of Tantalum Powder

In placement method C, where the materials are uniformly mixed, the reaction proceeds more homogeneously, and no intermediate phases are grown. This study further investigates the influence of temperature and time on the physical and chemical properties of tantalum powder synthesized under uniform mixing conditions (method C).

**Figure 12:**
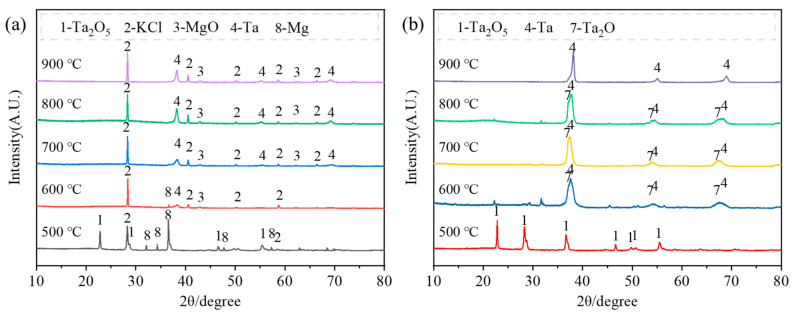
XRD patterns of the products: (**a**) before and (**b**) after pickling at different reduction temperatures.

Figure 12 illustrates the effect of temperature on phase transformation during the reduction process. The results indicate that no reaction occurs at 500 °C. However, at 600 °C, the tantalum phase begins to form in the system without any magnesium tantalates. A comparison of the phases before and after acid washing reveals that the diffraction peaks of the tantalum phase split into double peaks corresponding to Ta and Ta_2_O. This double peak effect becomes more pronounced at lower reduction temperatures, indicating incomplete deoxygenation. Due to the smaller particle size and higher surface activity, tantalum powder is more prone to oxygen absorption, resulting in Ta_2_O (with an oxygen mass fraction of 4.23%) formation during water washing. As the reduction temperature increases, the double peak phenomenon disappears, and only the tantalum phase remains in the washed products.

**Figure 13:**
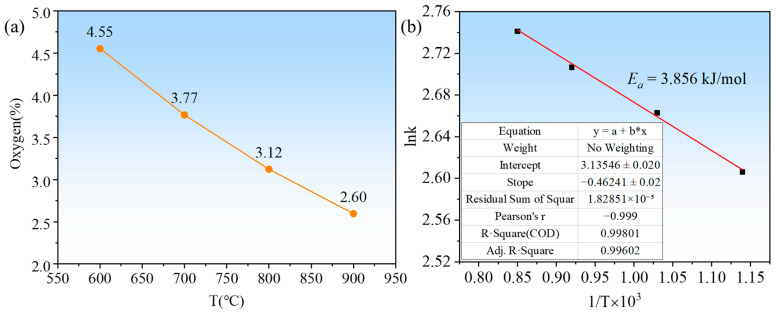
Change in oxygen content at different reaction temperatures (**a**); Plot of ln k vs. 1/T×10^3^ for the estimation of the activation energy of the reduced Ta powder (**b**).

Figure 13a shows the variation of oxygen content at different reaction temperatures. As can be seen from the figure, the oxygen content in the tantalum powder gradually decreases as the reaction temperature increases. The activation energy of the reaction was calculated from the change in oxygen content at different reaction temperatures using Equation (13), as shown in Figure 13b.

(13) $$k={\mathrm{Ae}}^{-E_{a}/(RT)}$$

where *k* denotes the reaction rate constant, A denotes the constant, R denotes the gas constant, *T* denotes the absolute temperature, and *E_a_* denotes the activation energy. The oxygen content in tantalum pentoxide is 18.1%, and assuming that the reduction in oxygen content corresponds to the reaction rate constant *k*, i.e., the value of *k* is proportional to the reduction in oxygen content, the reduction in oxygen content at each temperature is taken as the reaction rate constant *k*. From this, the activation energy of the reaction is calculated to be 3.856 kJ/mol. As the reaction is exothermic, the reaction itself releases heat, providing the molecules of the reactants with additional energy, making it easier for them to reach an active state and react chemically, resulting in a lower activation energy for the reaction.

**Figure 14:**
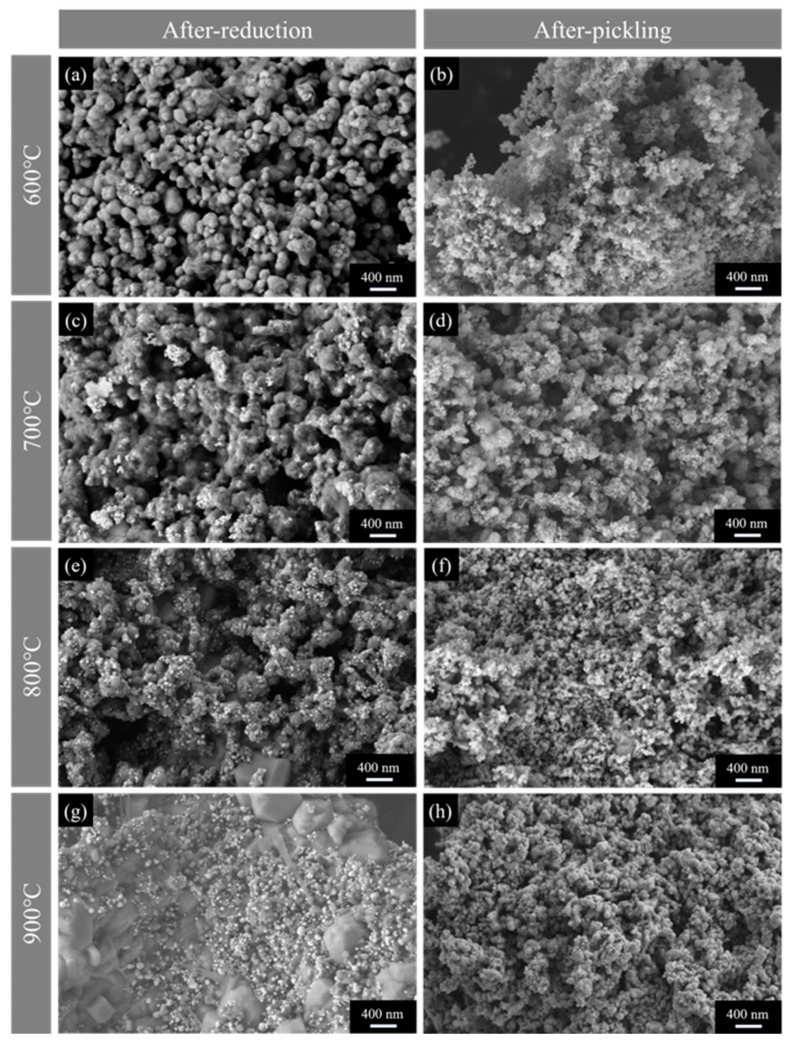
SEM images showing the morphology of the products: (**a**,**c**,**e**,**g**) before and (**b**,**d**,**f**,**h**) after pickling at different reduction temperatures.

Figure 14 shows the morphology of the products before and after acid washing synthesized under uniform mixing conditions (placement method C) at different reaction temperatures.

In Figure 14a,c,e,g, the interaction between magnesium and tantalum pentoxide initiates surface reactions and nucleation, forming tantalum powder particles. As the reaction temperature increases, both tantalum powder and magnesium oxide particles grow significantly. At 600 °C, fine tantalum powder adhering to the surface of magnesium oxide is formed. With a further increase in temperature, the number and size of tantalum powder particles increase markedly, with magnesium oxide particles growing more prominently into regular block shapes.

Figure 14b,d,f,h compares the morphology of the products after water washing. At 600 °C, the tantalum powder obtained is fine and weakly agglomerated, with a uniform morphology and a particle size of around 30 nm. As the reduction temperature increases, the tantalum powder particles grow significantly, reaching a size range of 50 to 150 nm at 900 °C. The synthesized particles of tantalum powder exhibit more regular shapes, better crystallinity, and improved dispersion. This improvement may be attributed to the decreased viscosity of the molten salt at higher temperatures, which reduces resistance to mass transfer and facilitates particle coalescence in the molten salt.

**Figure 15:**
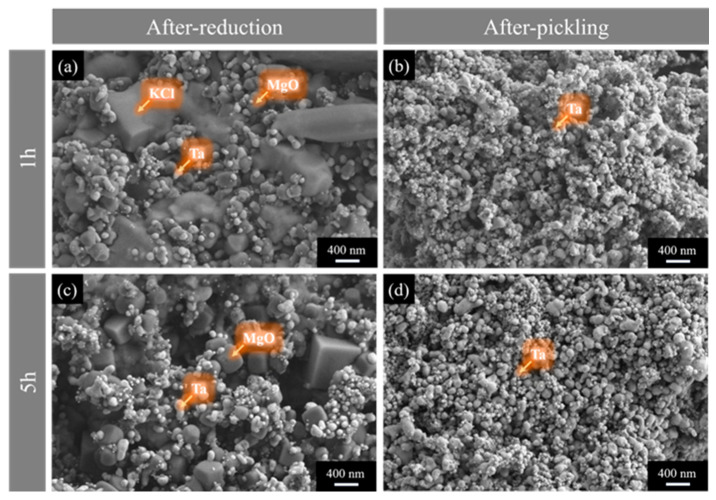
SEM images showing morphology of the products: (**a**,**c**) before and (**b**,**d**) after pickling at 900 °C for different holding times of (**a**,**b**) 1 h and (**c**,**d**) 5 h.

Figure 15 presents the microstructure of tantalum powder before and after washing with water at 900 °C with different holding times. As shown in Figure 15a,c, extending the holding time from 1 h to 5 h leads to significant growth in the magnesium oxide particles with good crystallinity. Figure 15b,d show that with longer holding times, the number of small particles in the tantalum powder decreases, resulting in enhanced uniformity. This indicates that regulating the temperature and the holding time contributes to the growth of tantalum powder with higher uniformity. Additionally, measurements of the oxygen content in tantalum powder synthesized at different holding times show that longer holding times help reduce the oxygen content. For instance, when held for 3 h, the oxygen content in the tantalum powder is 1.79%. Therefore, by carefully controlling the reaction temperature and holding time, it is possible to effectively regulate the oxygen content, uniformity, particle size, and particle size distribution of high-purity tantalum powder in the magnesium reduction reaction.

## 4. Conclusions

This study systematically investigated the phase transformation process during the magnesium reduction of tantalum pentoxide by varying material placement methods to adjust the reaction rate. The influence of different process parameters on the properties of tantalum powder was then studied under placement method C, based on the observed phase transformation patterns.

(1) During the magnesium reduction process, no lower-valence tantalum oxides were formed, consistent with thermodynamic calculations. However, in placement methods A and B, regions with insufficient magnesium were present during the reaction, where the by-product magnesium oxide was easily combined with tantalum pentoxide to form magnesium tantalate (MgTa_2_O_6_ and Mg_4_Ta_2_O_9_). As the reaction proceeded in magnesium-rich conditions, these magnesium tantalates (MgTa_2_O_6_ and Mg_4_Ta_2_O_9_) were further reduced to tantalum, resulting in multiple reaction pathways (Ta_2_O_5_ → Ta and Ta_2_O_5_ → MgTa*_x_*O*_y_* → Ta). In contrast, in placement method C, tantalum pentoxide was directly reduced to high-purity metallic tantalum, following a single reaction pathway (Ta_2_O_5_ → Ta).
(2) Tantalum powder obtained via the single pathway (placement method C) had a more concentrated particle size distribution, with fewer small particles and better particle uniformity, compared to the powder obtained via multiple pathways (placement method B). To ensure uniformity in tantalum powder particles, maintaining consistency in the reaction pathway during the reduction process is essential.
(3) Under placement method C, the magnesium reduction of Ta_2_O_5_ occurred only at temperatures above 600 °C. At lower temperatures, the resulting particles were finer and had higher activity, with increased oxygen content after water washing, resulting in the presence of Ta_2_O and Ta phases instead of pure Ta. At 900 °C, a pure tantalum phase was obtained. As the reduction temperature increased from 600 °C to 900 °C, the particle size of the tantalum powder gradually increased from 30 nm to 150 nm, but the particle uniformity decreased. However, extending the holding time improved the uniformity of the tantalum powder morphology, and the oxygen content in the tantalum powder decreased to 1.79% after a holding time of 3 h.
(4) The in-depth study of the phase transformation laws during the magnesium reduction of tantalum pentoxide and the influence of process parameters on the properties of tantalum powder will provide an important theoretical basis and technical support for the production and application of tantalum powder. In addition, this research can serve as a reference for studies on other metal reduction processes. Furthermore, by focusing on the magnesium tantalate formed during the reaction, our team aims to further synthesize a single phase of magnesium tantalate by solid-state synthesis, refine its thermodynamic parameters, and reduce it in order to further investigate its effect on the properties of tantalum powder.

## Figures and Tables

**Table 1:** Laser particle size variation of tantalum powder under different process conditions.

Placement Methods	Time (h)	*D*_10_ (μm)	*D*_50_ (μm)	*D*_90_ (μm)	*SPAN*
B	3	0.127	0.170	0.229	0.60
B	5	0.129	0.170	0.226	0.57
C	1	0.135	0.169	0.213	0.46
C	3	0.136	0.169	0.209	0.43

## Data Availability

The original contributions presented in this study are included in the article. Further inquiries can be directed to the corresponding author.
